# Applications of Infrared Thermography in Ophthalmology

**DOI:** 10.3390/life13030723

**Published:** 2023-03-08

**Authors:** Rosario Gulias-Cañizo, Maria Elisa Rodríguez-Malagón, Loubette Botello-González, Valeria Belden-Reyes, Francisco Amparo, Manuel Garza-Leon

**Affiliations:** 1 Centro de Investigación en Ciencias de la Salud, Universidad Anahuac México, Naucalpan de Juárez 52786, Mexico; 2 Division of Health Sciences, Department of Clinical Sciences, University of Monterrey, San Pedro Gaza García 66238, Mexico

**Keywords:** thermography, ocular surface temperature, dry eye, diagnostic methods

## Abstract

Body temperature is one of the key vital signs for determining a disease’s severity, as it reflects the thermal energy generated by an individual’s metabolism. Since the first study on the relationship between body temperature and diseases by Carl Reinhold August Wunderlich at the end of the 19th century, various forms of thermometers have been developed to measure body temperature. Traditionally, methods for measuring temperature can be invasive, semi-invasive, and non-invasive. In recent years, great technological advances have reduced the cost of thermographic cameras, which allowed extending their use. Thermal cameras capture the infrared radiation of the electromagnetic spectrum and process the images to represent the temperature of the object under study through a range of colors, where each color and its hue indicate a previously established temperature. Currently, cameras have a sensitivity that allows them to detect changes in temperature as small as 0.01 °C. Along with its use in other areas of medicine, thermography has been used at the ocular level for more than 50 years. In healthy subjects, the literature reports that the average corneal temperature ranges from 32.9 to 36 °C. One of the possible sources of variability in normal values is age, and other possible sources of variation are gender and external temperature. In addition to the evaluation of healthy subjects, thermography has been used to evaluate its usefulness in various eye diseases, such as Graves’ orbitopathy, and tear duct obstruction for orbital diseases. The ocular surface is the most studied area. Ocular surface temperature is influenced by multiple conditions, one of the most studied being dry eye; other diseases studied include allergic conjunctivitis and pterygium as well as systemic diseases such as carotid artery stenosis. Among the corneal diseases studied are keratoconus, infectious keratitis, corneal graft rejection, the use of scleral or soft contact lenses, and the response to refractive or cataract surgery. Other diseases where thermographic features have been reported are glaucoma, diabetic retinopathy, age-related macular degeneration, retinal vascular occlusions, intraocular tumors as well as scleritis, and other inflammatory eye diseases.

## 1. Introduction

The human body is homeothermic, which means that it maintains its temperature within a certain range to coordinate its metabolic activities, a task achieved through the body’s inherent thermoregulatory mechanisms [1]. Body temperature is one of the key vital signs for determining the severity of a disease, as it is a reflection of the thermal energy generated by an individual’s metabolism. The first study on the relationship between body temperature and diseases was carried out by Carl Reinhold August Wunderlich at the end of the 19th century, who stated that variations in body temperature should be considered significant since they occur due to a specific cause. Therefore, he considered body temperature as a means to confirm or rule out the existence of any disease [2].

During the last decades, various forms of thermometers have been developed to measure body temperature. Traditionally, methods for measuring temperature can be invasive, semi-invasive, and non-invasive. Non-invasive methods measure temperature in areas such as the armpit, ear canal, neck, chest, etc. Semi-invasive methods require the temperature transducer to be placed inside the body through a natural orifice without causing much discomfort (for example, oral temperature). Invasive methods require the transducer to be placed in a position deep in the body (for example, the rectum, vagina, esophagus, nasal cavity, digestive tract, or blood vessel).

Also, there are three heat transfer mechanisms on which thermometers are based to detect body temperature: conduction, convection, and radiation. The mechanisms of heat convection and radiation are used by infrared thermography, a technique [3] that, unlike the previously mentioned temperature measurements, allows one to observe and determine the temperature of any object or living being without direct contact with it [3].

The German astronomer and musician Sir Frederick Wiliam Herschel discovered infrared radiation empirically around 1800 [4]. After its discovery, this field began to evolve after 1900 when infrared radiation detection systems were developed. Its application began in the Second World War (1939–1945) when the scientists of the time tried to measure infrared radiation to identify and recognize enemy targets. The first images of infrared radiation documented in medicine were published in 1956 by LR Lawson who used thermography to analyze changes in temperature and vascularization caused by breast cancer [5], and in 1982 the US Food and Drug Administration (FDA) approved thermography as an adjuvant technique for the diagnosis of breast cancer [5]. As an example of its usefulness, a prospective clinical study published in 2008 evaluated 92 women with a previous suspicious mammogram or ultrasound result and demonstrated the precision and quality of the thermographic cameras, which detected malignant breast disorders with a sensitivity of 97% and a negative predictive value of 82% [6].

This technique has advanced and its theoretical basis has been strengthened, and it is now widely accepted and used in various disciplines, including medicine [7,8]. Initially, its application was limited by the low resolution of the images, the large size of the equipment, and the lack of computerized analytical tools. In recent years, great technological advances have reduced the cost of thermographic cameras, which allowed extending their use. Thermal cameras capture the infrared radiation of the electromagnetic spectrum and process the images to represent the temperature of the object under study through a range of colors, where each color and its hue indicate a previously established temperature [7]. Thus, because the human body loses excess heat mainly through radiation, the cameras measure the wavelength of the emitted infrared radiation and calculate its temperature [3] so that when physiological or pathological changes occur in any part of the body, the thermal balance is affected and the temperature of tissue increases or decreases compared to the adjacent tissue, allowing thermography to identify not only the location but also the extent of an injury [7]. In addition, a significant evolution has been achieved so that its application is increasingly sensitive and specific. Another advance has been the precision of the detection of temperature change since in the past, thermographic cameras were only capable of detecting temperature changes of 0.5–1 °C [6]. Currently, cameras have a sensitivity that allows them to detect changes in temperature as small as 0.01 °C [9].

It is important to mention that several variables can influence body temperature such as age, sex, body mass index (BMI), and environmental conditions (temperature, humidity, light, etc.) of the location where the images are captured [10]. Therefore, body temperature must be evaluated considering the variability of each individual, that is, a baseline value must always be taken in the same location so that results are reproducible [11]. The main advantage of thermography is that it is a fast, non-invasive technique that does not emit ionizing radiation, and the images it produces are easy to interpret. The main drawback is its vulnerability to the patient’s intrinsic and extrinsic variables [6].

Several research studies have been published on different medical applications of thermography: to diagnose, localize and evaluate the response to treatment of acute chronic pain [12], monitor the impact of a stroke rehabilitation program [13], for the identification and monitoring of Raynaud’s phenomenon and other vasospastic disorders, for the prevention of diabetic foot ulceration, for the monitoring of infections, the objective measurement of the intensity and extent of joint inflammation in rheumatoid arthritis [8], and for preoperative planning and assessment of free flap perfusion after vascular connection [3], among others. With the appearance of the COVID-19 pandemic, the use of temperature measurement as a screening method was proposed in the first months of the pandemic; however, there are conflicting results about its value. Khaksari et al. found in a literature review that studies evaluating IRT showed varied results regarding its efficacy for the screening of infectious diseases. This suggests the need to assess additional physiological parameters to increase the sensitivity and specificity of non-invasive biosensors [14]. However, more recently, Makino Antunes et al. reported a predictive model that reached an accuracy of 86% for disease detection [15]. In addition to the traditional measurement of temperature by infrared methods, there have been reports of algorithms based on artificial intelligence and the use of drones with thermal cameras for temperature measurement and algorithms to recognize human actions such as coughing and sneezing, which are paramount symptoms of respiratory infections [16].

The objective of this review is to evaluate the applications of thermography in ophthalmology. To exemplify possible temperature changes associated with some of the clinical situations described, the article includes images taken by the authors with a FLIR A70 camera (IR resolution: 640 × 480 pixels, thermal sensitivity/NETD: 35 Mk (0.035 °C), accuracy of ±2 °C in a measuring range from −20 to 175 °C, frequency: 30 Hz, 29° × 22° optics (14.3 mm) with manual focus) with the bcbThermoPro software (bcbMonitor4.0 V102) provided by Bcb Engineering company (Apodaca, Nuevo León, México). To take the images, all subjects spent at least 30 min in a room with a temperature of 25 °C to reduce the effect of ambient temperature on the results.

### Method of Literature Search

We performed a search on 1 March 2022, in PubMed (all fields), Embase (abstract, title), and Lilacs (title, abstract, keyword) databases to identify all published studies using the following search strategy: (thermography AND eye) and (ocular thermography). No limits were used for the publication date. We screened the retrieved articles first by title and abstract and subsequently by full text. We checked all references to detect studies not found using our original database search. We included papers in German, English, or Spanish.

## 2. Use of Thermography in Ophthalmology

Along with its use in other areas of medicine, thermography has been used at the ocular level for more than 50 years, when Schwartz studied the temperature of the ocular surface, conjunctiva, and cornea of rabbits using a string thermistor, observing a direct relationship between environmental humidity and temperature with ocular parameters [17]. Since then, various techniques for measuring ocular temperature have been described [18]. The advancement of technology for infrared temperature measurement has allowed the use of thermography in various ophthalmology subspecialties both for diagnostic purposes and for monitoring surgical procedures and tissue responses, as described below.

Infrared ocular thermography determines the ocular surface temperature (OST) by measuring the amount of infrared radiation emitted from the surface with an infrared thermal imaging camera [19]. It is a non-invasive dynamic technique that offers a perspective of ocular physiology and the characteristics of tissue metabolism [20].

Tissue temperature is influenced by several external and internal factors [21,22,23]; at the corneal level, the influence of these factors has also been demonstrated since the initial studies by Schwartz [17]. More recently, the effect of body and environmental temperature on corneal temperature has been studied. Kessel et al. found that although there is a linear relationship between both temperatures, from 36.5 to 37 °C there is a stabilization of corneal temperature despite the increase in body temperature; this stability is also observed when modifying environmental temperature up to 34.5 °C [24]. This relationship is also found on the ocular surface; Tan et al. found that a 1 °C increase in ambient temperature can cause a 0.15 °C to 0.2 °C increase in OST [25]. Purslow et al. found that for every 1 °C increase in body temperature, there is an almost identical increase in OST [20]; this also occurs when ambient temperature decreases, although less proportionally, since lowering the temperature from 25 to 5 °C produces a reduction in OST of only 4 °C [26]. The most important internal factor for maintaining OST is ocular vascular tone; Vannetti et al. showed that peripheral vasoconstriction increases OST (increasing range 0.1 to 0.2 °C) due to tear film evaporation [27].

Additionally, there is also a difference in the temperature of the different regions of the ocular surface. Matteoli et al. found that the nasal canthus is the warmest region, and the central cornea is the coldest; this temperature distribution is mainly due to blood perfusion [16]. It is now recognized that the highest temperature of the nasal canthus is due to the supraorbital artery, a direct branch of the ocular artery [28]. In contrast, the central cornea shows the coldest temperature because it is anatomically avascular and its temperature depends mainly on tear film distribution and environmental conditions [22]. There are also variations in the corneal regions, as shown by Purslow et al., who found a temperature increase of 0.45 and 1.0 °C when comparing the limbus with the central cornea [18]. Moreover, the cornea is warmer right after blinking because it is warmed by the passage of the upper eyelid [29], but the longer the eye stays open, the more the ocular surface cools as heat is transferred to the environment during tear film evaporation [26].

Finally, in people without unilateral systemic vascular diseases, no significant differences have been found between the right and left eyes [22].

### 2.1. Thermographic Characteristics in Healthy People

**Figure 1:**
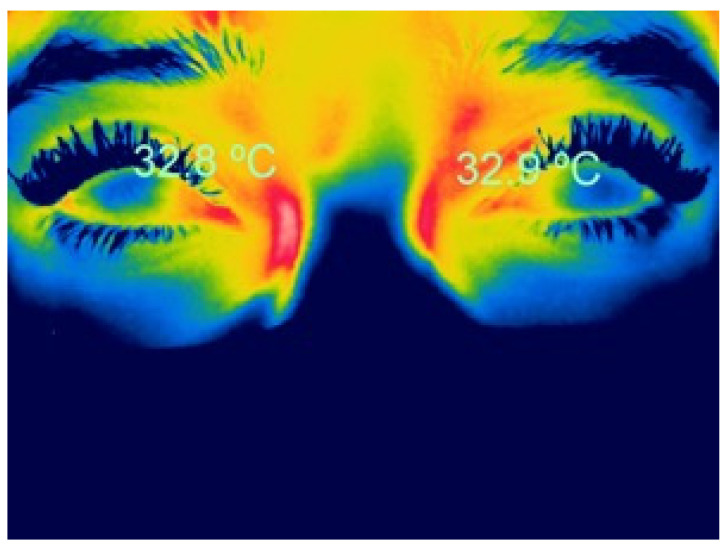
Infrared thermographic image of corneal temperature in a healthy woman.

Initially, ocular thermography was performed using contact techniques, often invasive, which recorded a highly variable mean corneal temperature (35.9 ± 0.7 °C) due to the different methods used and variable ocular measurement zones [18]. With current techniques, the literature reports that the average corneal temperature ranges from 32.9 to 36 °C [22] (Figure 1). Recently, a study of dynamic ocular surface temperature measurement in healthy subjects with a new thermographic device (Tomey TG 1000, Tomey Corp., Nagoya, Aichi, Japan) found a mean ocular surface temperature of 34.02 ± 0.22 °C [30].

One of the possible sources of variability in normal values is age. Acharya and colleagues reported a variation of almost two degrees between young patients (19 ± 9 years) and older adults (61 ± 11 years) as well as a regional variation in the corneal temperature [21]. Other studies have found a trend of decreased OST with age, of between −0.010 °C and −0.02 °C per year in healthy people [23]. Finally, the temperature of the cornea and the ocular surface can vary as a consequence of tear evaporation due to a longer exposure time to the environment secondary to a low blink rate or keeping the eyelids open.

To avoid bias in OST measurement in patients, it is important to consider that the temperature should be taken at the same time, in a specific room with a set room temperature (uniform) and without drafts. In addition, to avoid the influence of external temperature, it is recommended that the participants stay in a room with a stable temperature for 15 to 20 min before taking any measurements.

### 2.2. Use of Thermography in Ocular Disorders

#### 2.2.1. Orbit, Eyelids, and Tear Ducts

**Figure 2:**
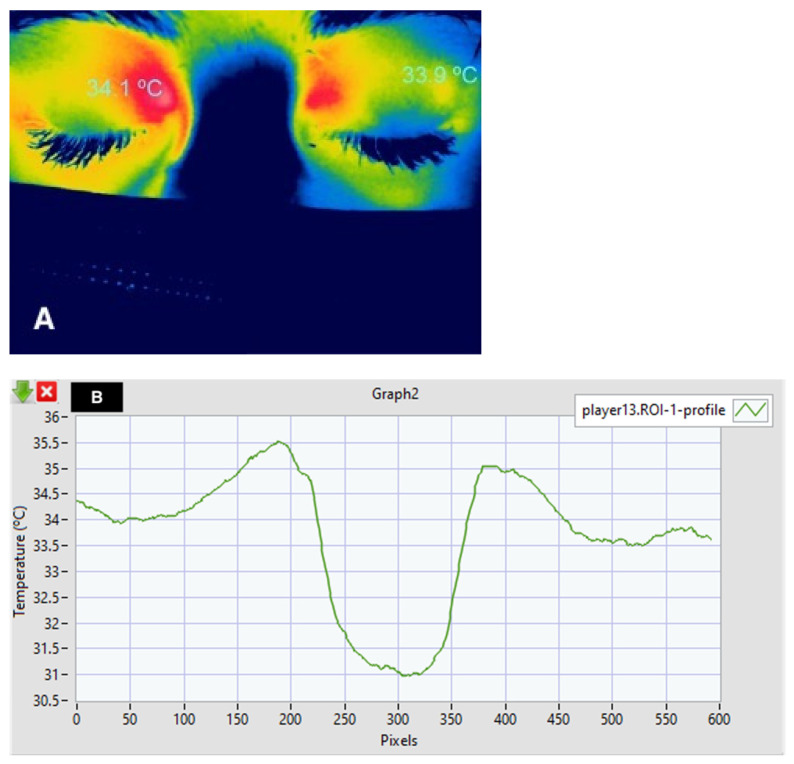
Thermography of the face of a healthy patient wearing a mask, showing the lowest temperature of the nasal bridge and the highest temperature in the upper region of the internal canthus (**A**), and a graph of the thermographic changes along the horizontal axis (**B**).

With the use of thermography, it is possible to evaluate the temperature of the entire orbital region, as in the cornea, the temperature can vary according to the different regions, probably associated with differences in the vascularization of the region (Figure 2A,B). Kholman et al. were the first to describe the use of thermographic images for the diagnosis of orbital disease [31].

Graves orbitopathy (GO) is the most common extrathyroidal manifestation of Graves’ disease [32]. It is characterized by two phases, an initial inflammatory phase characterized by infiltration of the orbital tissues predominantly by CD4+ and CD8+ T lymphocytes and B lymphocytes [33], and another inactive phase with scarring predominance [32]. Several studies have evaluated thermographic analysis for the diagnosis [34,35], monitoring of therapeutic response in the inflammatory phase [34,36], and prognostic purposes in response to treatment [37]. Dave et al. studied 97 eyes of 77 patients (11 eyes of 11 patients with active GO, 46 eyes of 46 patients with inactive GO, and 40 eyes of 20 healthy subjects), finding that temperature in the caruncle and medial and temporal conjunctiva in the active GO group was uniformly and significantly higher than the corresponding area in the inactive GO and healthy groups. The eyelids and cornea did not show higher temperatures in cases of active GO compared to the other two groups [35]. Minatel et al. obtained similar results when studying 198 patients, measuring the temperature in the region of the caruncle and the upper eyelid. Twelve patients (6.06%) had active GO, 62 (31.31%) had inactive GO, 62 (31.31%) had Graves’ disease without ophthalmopathy, and 62 (31.31%) were healthy controls. Patients with active GO had higher temperatures in all ocular areas compared to the other groups (*p* < 0.0001). In addition, there was a positive correlation with the clinical activity score. No significant differences in temperature were observed between patients with inactive GO, patients with Graves’ disease without apparent eye disease, and healthy subjects [36]. Chang et al. corroborated the thermographic differences between healthy subjects and patients with active GO. Furthermore, they observed that the temperature of the caruncle, medial and lateral conjunctiva, and lower eyelid of patients with active GO was higher than in healthy subjects; they observed no differences in the upper eyelid, cornea, and lateral orbit temperatures between patients and healthy controls [34]. When treating patients with GO with pulses of intravenous methylprednisolone, the temperatures of the caruncle, medial conjunctiva, and lower eyelid decreased significantly. The sum of the temperatures of the upper lid, caruncle, medial and lateral conjunctiva, lower lid, and cornea also decreased significantly after treatment, The sum of the temperatures of the upper eyelid, caruncle, medial conjunctiva, lateral conjunctiva, lower eyelid, and cornea also significantly decreased after treatment (201.9 ± 7.0 °C vs. 196.9 ± 9.2 °C, *p* < 0.05) [34]. Another study reported similar findings in patients with active GO treated with a bolus of intravenous methylprednisolone. Multivariate analysis showed that pre-treatment temperature was negatively correlated with the mean reduction in post-treatment temperature. In addition, patients with higher disease activity, and higher temperature before treatment, were more likely to improve on the clinical activity scale after treatment [37].

Another application of thermography is its use for the diagnosis of tear duct obstruction. Machado et al. reported the thermographic findings in a 56-year-old woman with acute chronic dacryocystitis and obstruction of the upper and lower lacrimal points, finding hyporadiation in the area corresponding to dilation of the lacrimal sac of the affected eye, which could be associated with the degree of edema caused by tissue inflammation. In addition, this thermographic dynamic study showed no cooling of the lacrimal sac of the affected eye when applying cold drops, while the temperature decreased on the contralateral side with a healthy, open tear duct [38].

#### 2.2.2. Ocular Surface

Ocular surface temperature is influenced by multiple conditions, one of the most studied being dry eye [39,40], which will be discussed in a special section. Due to the inflammatory and vascular processes associated with allergic conjunctivitis, it is possible to identify thermographic changes in the ocular surface in patients with allergic conjunctivitis. Hara et al. reported an increase in basal OS temperature when exposing the eyes to a conjunctival allergen; however, eyes treated with antihistamines showed a smaller increase in temperature compared to eyes treated with lubricants; a correlation was also observed between increased temperature and conjunctival hyperemia, but not with pruritus and chemosis [41].

Another ocular surface disorder studied by thermography is pterygium. Gonnermann et al. studied pterygium patients with dry eye and healthy subjects and did not find a difference in ocular surface temperature between both groups (*p* = 0.551), although they observed a lower temperature in patients with pterygium compared with patients with dry eye (*p* = 0.003) [42].

The measurement of ocular surface temperature can also reflect alterations at the systemic level. Morgan et al. evaluated patients with carotid artery stenosis (CAS) by Doppler ultrasonography and infrared thermography and found a negative correlation between the OST and CAS grade (r = −0.67, *p* < 0.001) [43], showing that the degree of blood supply to the ocular surface modifies the temperature. This was supported by the experiment of Vanneti et al. who measured ocular surface temperature before and after inducing peripheral vasoconstriction, finding that ocular surface temperature increased significantly (*p* < 0.05), especially near sources of ocular blood supply, that is, the temporal and nasal areas [27].

Please see Table 1 for a comprehensive summary illustrating relevant OST values and the room temperature at which they were measured.

#### 2.2.3. Cornea

The cornea is one of the eye tissues whose temperature depends on other components such as tear film (TF) evaporation, so after each blink, TF redistribution causes changes in corneal temperature. Furthermore, because the cornea is avascular, the corneal central temperature depends on the conduction and convection of heat by the aqueous humor, whose temperature depends, in turn, on the blood flow of the ciliary body. Peripheral corneal temperature is influenced by the blood flow of the perilimbal vessels.

Mapstone introduced the measurement of OST through radiation (non-contact) thermography in 1968, and studied various thermal patterns of structures such as the cornea and periorbital skin and became a reference in this technique since previous corneal temperature studies used contact methods [47,48,49]. Another of the first studies (1999) that evaluated corneal temperature showed a decrease of less than 0.010 °C per year of age, being more evident after 50 years, something that is related to the age-associated decrease in blood supply to the eye [23].

Other studies have focused on optimizing and standardizing the measurement of corneal central temperature, reducing the influence of artifacts to evaluate the reproducibility of the measurements, and observing that the values are reproducible over 2 weeks and that they are dependent on body temperature [50]. It has also been reported that there is an initial linear increase in corneal temperature related to the increase in body temperature, but corneal temperature stabilizes between 36.5 °C and 37.0 °C, despite a continuous increase in central body temperature [24].

**Figure 3:**
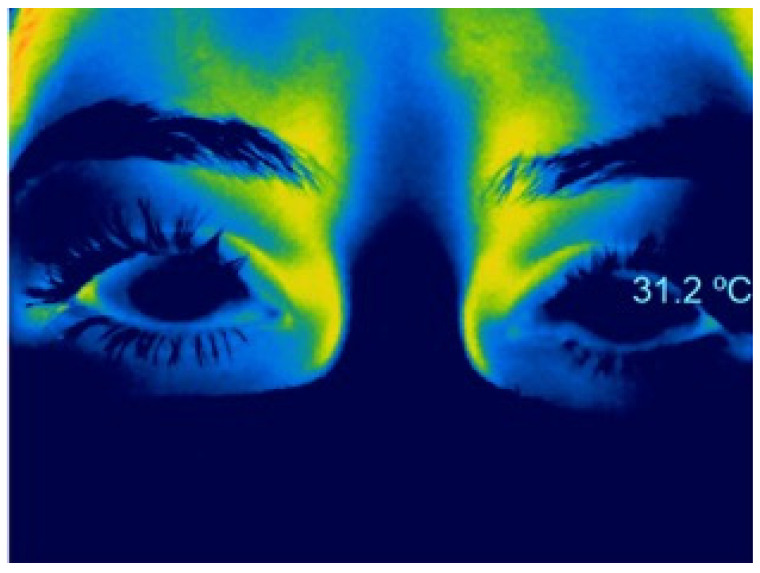
Thermographic image of a patient after 6 h of wearing a soft contact lens in both eyes.

**Figure 4:**
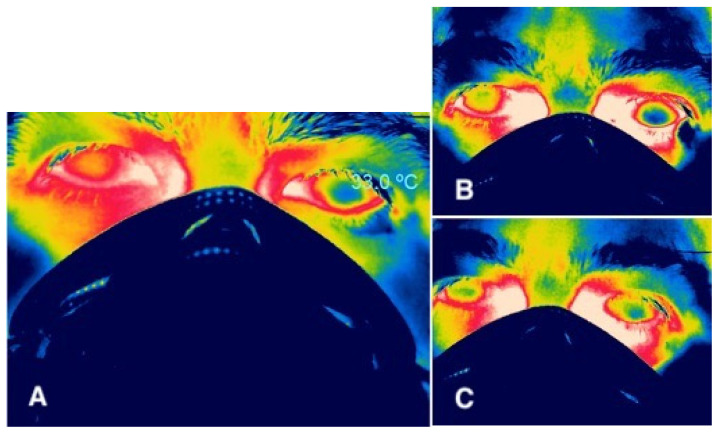
Thermographic image of a patient who has a soft contact lens placed in the left eye, resulting in a drop in corneal temperature (**A**), and an increase in temperature after contact lens removal, immediately after removal (**B**), and at 2 min (**C**). All images used the same color scale to show temperature variations.

Since keratoconus has been identified as an inflammatory disease, several studies have evaluated OST in patients with keratoconus, without finding an increase [51]. The relationship between endothelial cell density and central OST was also evaluated in healthy subjects and patients with keratoconus, observing a weak correlation between OST in the central cornea with endothelial cell density both in normal subjects and in patients with keratoconus. Another study evaluated the relationship between corneal thickness, endothelial cell density, and anterior chamber depth with OST, without finding a significant correlation in healthy subjects. However, the authors reported that the temperature ranges in the center of the cornea are higher and decrease as the cornea thins at the periphery [52]. It has also been evaluated whether the use of scleral contact lenses in patients with keratoconus modifies the temperature of the ocular surface, finding that there are no thermal modifications despite the accumulation of tears under the lens [53]. There is no consensus regarding CL effects on ocular surface temperature. The effect can vary due to several factors: the type of CL (soft, rigid gas-permeable, scleral) [54], as well as the material they are made of (hydrogel, polymethylmethacrylate) [55], and the amount of water they can absorb [56], among others. Carracedo et al. reported that the use of scleral CL in patients with keratoconus modifies OST, observing no thermal modifications despite the accumulation of tears under the lens [53]. Martin et al. reported similar findings with the use of soft CL in healthy young subjects [56]. In a yet unpublished study done by our group, we found that the use of soft CL decreases corneal temperature (Figure 3 and Figure 4), and these changes are reversed after removal both immediately (Figure 4B) and at 2 min (Figure 4C). This could be due to a real lower temperature of the prelens tear film in soft lens wearers compared to that of the non–CL-wearing eye [57], although the temperature of the postlens tear film is higher [53]. This difference between pre- and postlens tear film temperatures could be reflected in OST, or the observed OST changes may be due to the fact that the emissivity of the CL is not equivalent to that of the cornea e = 0.98 [58].

The relationship of corneal temperature with surgical procedures has also been evaluated; Betnek et al. reported a temperature increase during surface surgery with an excimer laser, although this increase in temperature does not produce denaturation of corneal collagen [59]. Another study showed that two different types of excimer lasers generate different increases in corneal temperature during use. In addition, this study reported that placing the thermography camera above the cornea ensures more accurate measurements since the camera is parallel to the radiation-emitting surface of the cornea, not tilted at 45° as has been done in other studies [60].

**Figure 5:**
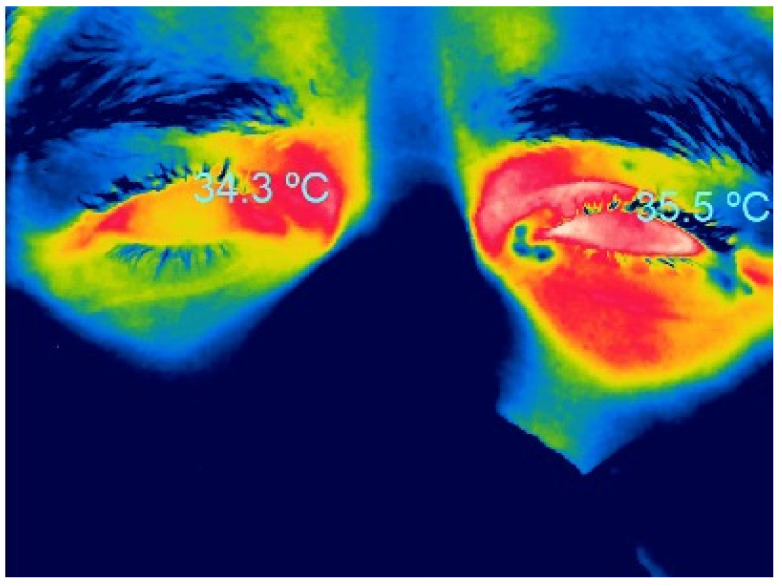
Thermographic image of a patient with a fungal corneal ulcer in the left eye that produced an increase in corneal temperature of 1.2 °C compared to the healthy eye and also in the lower palpebral region.

Finally, other studies have found that corneal temperature increases in cases of bacterial corneal ulcers, and that the severity of the infection is directly proportional to the temperature measured, that is, the higher the severity, the higher the temperature (Figure 5). This finding raises the possibility of using thermography to objectively measure inflammation in ulcer cases, an aspect that is difficult to assess by clinical examination alone [61]. The same happens in patients with corneal transplant rejection, which sometimes cannot be diagnosed in the early stages. Sniegowski et al. compared a patient with acute corneal rejection with healthy eyes, and observed a significant increase in OST in the affected eye compared to the fellow eye of the patient and the rest of the healthy eyes, concluding that ocular thermography is a complementary diagnostic tool that may be useful for routine monitoring of corneal transplant patients [62].

#### 2.2.4. Dry Eye Disease

Dry eye disease (DED) is a multifactorial disease in which a defined dysfunction or deficiency of the tear film is observed, accompanied by ocular clinical manifestations; in this condition, there is a loss of tear film homeostasis with hyperosmolarity and inflammation of the ocular surface [63].

The prevalence of dry eye is estimated to be between 5 and 50% of the population, with evaporative dry eye being the most frequent [64]. The National Survey of Health and Welfare reported that in the United States, 6.8% of the population suffers from dry eye, and this prevalence is significantly modified by age since it was found that in young adults aged 18–34 years, the prevalence was 2.7% while in people older than 75 years the prevalence was 18.6% [65].

The approach for dry eye diagnosis includes several tests such as questionnaires, ocular surface staining, tear film break-up time (TFBUT), and other auxiliary methods such as the Schirmer test and meibography. The most widely used approach is that described in the DEWS II report, whose diagnostic criteria are a score of 13 points or higher in the ocular surface index questionnaire, a measurement of <10 mm/5 min in the Schirmer test, and a TFBUT of less than 10 s [66].

In recent years, the number of reports on the use of thermography as an auxiliary diagnostic method for dry eye has increased [19,26,39,44,45,67,68,69,70,71,72,73].

This is based on the correlation between OST and dry eye diagnostic parameters (such as TFBUT) [26,74] and well-established pathophysiological mechanisms of dry eye disease. Giraldez et al. demonstrated that there is a relationship between tear film thickness and OST measured with infrared thermography [70], where the higher the OST, the greater the tear film thickness values observed. This phenomenon can be explained by understanding that the tear film can act as an insulator that retains heat. Furthermore, in dry eye patients, there is a direct relationship between OST and tear film stability, where OST is inversely proportional to the rate of evaporation [75]. Most studies report that dry eye patients have lower OST than healthy patients [26,71,74,76]. However, Morgan et al. found that mean OST was significantly higher in the dry eye group than in controls (*p* < 0.01) [72]; this difference could be because the technology used at the time to measure temperature was infrared radiation, while currently, the use of infrared thermography prevails. In addition, other studies did not observe a difference in temperature between dry eye and healthy subjects [29,45,71], which again could be due to the technology used (infrared radiation) or because only the central corneal temperature was measured; likewise, there is controversy about the most appropriate region to discriminate between patients with dry eye vs. healthy subjects. Initially, it was considered that the central corneal region was the most important [77]; however, Zhang et al. recently studied patients with evaporative DED and, although they found that all regions (except the inferior corneal region) showed higher temperatures in patients with dry eye, the region with the best diagnostic performance was the nasal region and best correlation with the severity of DED [39]. In addition, the difference between the central corneal temperature and the sclerocorneal limbus temperature is greater in patients with dry eye than in healthy controls [72].

Another widely studied feature is the behavior of OST when keeping the eyes open. Various reports in the literature confirm that patients with dry eye show a drop in temperature when keeping the eyelids open [44,45,67,68,71,74,76,78,79], and these changes are significant from two seconds after the opening [72].

In addition to its use for diagnosing DED, thermography helps differentiate the dry eye subtype in patients. Zhang et al. reported that subjects with meibomian gland dysfunction (MGD) have a higher baseline temperature (*p* < 0.022) and a higher asymptotic temperature (*p* < 0.007) than subjects with aqueous-deficient dry eye [40], a similar result to the one reported by Abreau et al. [26], as shown in Table 2. It has also been reported that patients with evaporative dry eye have a higher cooling rate than patients with aqueous-deficient dry eye [44,79].

#### 2.2.5. Lens

**Figure 6:**
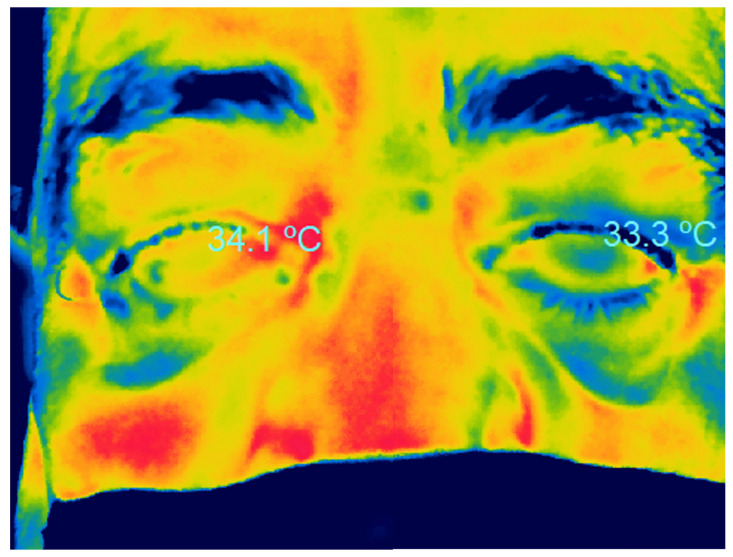
Thermographic image of a patient 24 h after phacoemulsification surgery and vitrectomy of the right eye; a difference of 0.8 °C is observed between the operated eye and the healthy eye.

The negative impact of dry eye on the surgical outcome of cataracts has been widely described [80,81,82,83]. Several studies have shown an increase in the signs and symptoms of dry eye after cataract surgery [84,85,86,87,88,89,90]. A recent meta-analysis found that patients with prior MGD are at increased risk of postoperative dry eye [87]. In addition to visual changes, dry eye after cataract surgery produces corneal thermographic changes. Giannaccare et al. studied patients that underwent uncomplicated phacoemulsification and found that after 7 days, there was a significant decrease in temperature both in the central 4 mm of the cornea and in the 4 mm of the nasal and temporal limbal corneal sector compared to preoperative values, with a partial recovery at 28 postoperative days. In addition to these thermographic changes, it was found that a higher temperature when opening the eye and 10 s later was associated with a higher score on the OSDI questionnaire and a shorter TFBUT. There is also a report on the correlation between the higher temperature in the temporal corneal sector and a greater inflammation in the anterior chamber as well as the concentration of albumin in the tear film, but without finding a correlation between the thermographic changes and the surgical parameters [91] (Figure 6). Modrzejewska et al. also found a decrease in corneal and orbital temperature 14 days after cataract surgery with a recovery to preoperative levels at 28 days [92]. These changes do not seem to be associated with the presence of the intraocular lens, since Sniegowski et al. studied patients with cataracts, a clear lens, and pseudophakic, and observed no differences in corneal temperature between the three groups [93].

Another application of thermography in cataract surgery has been for monitoring anterior chamber inflammation. Fujishima et al. evaluated patients who underwent cataract surgery using a manual technique and evaluated thermographic and inflammatory changes in the anterior chamber, comparing patients with surgeries that lasted more or less than 40 min, observing that only patients whose surgery lasted more than 40 min showed a rise in corneal temperature without observing a relationship between thermographic changes and the severity of anterior chamber inflammation [94].

#### 2.2.6. Glaucoma

Because neuroinflammation and the presence of immune responses have been implicated as causes of optic nerve damage in glaucoma, several studies have been conducted evaluating changes in OST in various subtypes of this disease, such as primary open-angle glaucoma (POAG) and pseudoexfoliation glaucoma, finding conflicting data. One study reported an increase in OST in patients with glaucoma [95], and many others reported a decrease, as explained below. There is also controversy regarding the relationship between corneal temperature and intraocular pressure (IOP) in patients with glaucoma since a study in healthy subjects showed that induced changes in corneal temperature (by increasing temperature after closing the eyelids for one hour) caused an IOP increase, and on the contrary, the use of a cooling mask induced a decrease in IOP that correlated with the decrease in corneal temperature [96]. Similarly, thermography provided indirect information about the influence of glaucoma medications; specifically, some antiglaucoma drugs have been shown to lower central corneal temperature at a time point coincident with their peak effect [97]; this supports the idea that OST may be elevated in patients with glaucoma.

However, as previously mentioned, the rest of the evidence supports a decrease in OST in glaucoma patients. The dynamics of OST in glaucoma subjects compared to healthy subjects have been evaluated, finding that subjects with glaucoma showed significantly lower temperatures in the central cornea immediately after opening the eye compared to the control group and, additionally, eyes with glaucoma cooled significantly faster (average decrease of 0.49 °C during the first second versus 0.24 °C in the control group) [46].

Other very comprehensive studies have exhaustively evaluated retrobulbar hemodynamics in patients with POAG using Doppler ultrasound of the ophthalmic artery, central retinal artery, and posterior short ciliary arteries, to determine if there was any correlation with OST measured by thermography compared to healthy controls. The results showed that although OST does not correlate with IOP, it does correlate with some Doppler parameters, specifically with higher values of the resistivity index in all retrobulbar vessels and lower values of end-diastolic velocity in the ophthalmic artery and the short posterior ciliary arteries, which means that OST could be used as an indirect marker of inadequate perfusion of the optic nerve head [98,99].

Thermography has also evaluated the function of the filtering bleb after trabeculectomy, observing that blebs in which the aqueous humor circulates towards the subconjunctival space, producing a decrease in intraocular pressure (functional blebs), have lower temperatures than those that are due to fibrosis of the tissue; a barrier is formed that does not allow the circulation of the aqueous humor (non-functional) ones because the aqueous humor is colder than the surrounding tissue, causing the corneal and filtering bleb temperature to drop to a level lower than that of the surrounding conjunctiva [100,101]; these results support the evidence showing lower corneal temperature in patients with glaucoma.

#### 2.2.7. Retina

Diabetes mellitus (DM) is one of the most common vascular diseases and affects a large number of patients at the level of the retinal vasculature, with perfusion and ischemia alterations. Two studies have evaluated OST in patients with diabetic retinopathy, finding a decrease in OST [102,103], although it has not been possible to establish a temperature threshold that allows discriminating between proliferative (PDR) and non-proliferative diabetic retinopathy (NPDR) [104].

Another of the most prevalent pathologies is age-related macular degeneration (AMD), whose thermographic results are controversial. On the one hand, a study investigated the OST of AMD patients vs. age-matched controls and found that OST was significantly lower in AMD patients [105] postulating, like other authors, a possible ischemic role in the pathophysiology of AMD [79,106]. There is also evidence of decreased OST in another ischemic disorder, central retinal vein occlusion (CRVO), associated with lower temperatures in the affected eye [107]. In contrast, in a recent study, AMD patients had elevated OST, and the authors postulate that this is due to the underlying inflammatory pathophysiology [103]. A study that may provide interesting data about this controversy evaluated OST in retinal arterial disorders on the basis that at the time the study was carried out, it was difficult to make a clinical distinction between an embolism of the central retinal artery and temporal arteritis and found that in temporal arteritis the corneal temperature is very low and there is a significant difference compared to the contralateral side. On the contrary, in central retinal artery (RCA) occlusion, the temperature of the affected eye shows a slight increase, possibly because choroidal circulation is not affected in this pathology and there may be choroidal vasodilation to compensate for retinal hypoxia [108]. In our opinion, there is probably a similar phenomenon of ischemia in AMD, supported by the results of the previously mentioned studies, but perhaps it is compensated by choroidal vasodilation as in RCA occlusion, as mentioned by Horven in his study [108]. These factors should be assessed in a randomized study including patients with different years since diagnosis of AMD as well as with all AMD subtypes, with or without the presence of concomitant conditions.

Finally, thermography has also been useful for the diagnosis of intraocular tumors [109], and its convenience in vitreoretinal surgery has been postulated. Since there are no recommendations on the ideal temperatures during this type of surgery, a recent study evaluated the epibulbar and intraocular temperature in patients undergoing vitreoretinal surgery for rhegmatogenous retinal detachment (RRD) and PDR, finding that during irrigation with saline solution at room temperature, there is a decrease in the temperature of the vitreous cavity, but when the irrigation is stopped, the vitreous cavity quickly recovers its baseline temperature. The authors also report hyperthermia after surgery (Figure 6) [110]. This study provides novel information that is not part of the routine intraoperative evaluation, but which the authors nonetheless propose as a method that would improve outcomes for patients.

#### 2.2.8. Inflammatory Ocular Diseases

Scleritides are a group of diseases in which there is an increase in the thickness of the episcleral blood vessels, which determines its presentation as a red eye, so the increase in blood flow must produce an increase in temperature [111]; however, there is little information in the literature in this regard. Kawali recently studied a series of cases that included patients with scleritis and found a higher temperature in patients with scleritis when compared to the healthy eye, the difference being greater when the scleritis was anterior compared to posterior scleritis and in a patient with postoperative anterior uveitis [112]. The same authors reported a higher temperature in the affected eye in patients with posterior scleritis compared to the healthy eye, with a reduction in temperature when initiating treatment [113].

In 1968, Mapstone evaluated the temperature of the cornea and the periorbital skin of a group of patients with anterior uveitis through a radiometric technique that uses a bolometer [48] and found that most patients had higher corneal and periorbital skin temperatures and that there was a linear relationship between corneal temperature and the degree and duration of ciliary injection [114].

## 3. Conclusions

Thermography seems to be a useful and underutilized technique in ophthalmology since it represents an easy-to-use and non-invasive portable modality that can be used in various pathologies of the different subspecialties. The single time-point OST of healthy eyes typically ranges from 34 to 36 °C.

## Figures and Tables

**Table 1:** Summary of OST and room temperature values.

Authors	Ambient Temperature	Relative Humidity	Ocular Surface Temperature
Kessel L, Johnson L, Arvidsson H, Larsen M [24]	32.0–34.5 °C	NR	36.5 °C–37 °C.
Tan JH, Ng EYK, Rajendra Acharya U, Chee C [25]	An increase of 1 °C	NR	Shows an increase of 0.15–0.2 °C in ocular surface temperature.
Purslow C, Wolffsohn J [20]	21.0 °C ± 0.6 °C	40% ± 2%	For every 1 °C rise in body temperature, there is almost an identical rise in OST.
Vannetti F, Sodi A, Lacarbonara F, Corvi A [27]	27.2 °C ± 0.4 °C	43% ± 4%	36.2 °C–37.7 °C.
Matteoli S, Vannetti F, Sodi A, Corvi A [22]	24.6 ± 2.1 °C	56% ± 8%	Nasal canthus: -Female 35.68 ± 0.49 °C. -Male 35.78 ± 0.49 °C. Central cornea: -Female 34.93 ± 0.46 °C. -Male 35.0 ± 0.48 °C.
Mori A, Oguchi Y, Okusawa Y, Ono M, Fujishima H, Tsubota K [29]	25.5 ± 0.5 °C	30% ± 6%	Normal patient: Temperature right after blink: 35.5 °C. Temperature 10 s after blinking: 34.5 °C. Dry eye patient: Temperature right after blinking: 35.3 °C. Temperature 10 s after blinking: 35.0 °C.
Abreau K, Callan C, Kottaiyan R, Zhang A, Yoon G, Aquavella JV, Zavislan J, Hindman HB [26]	24 °C ± 2 °C	40% ± 3%	Mean Initial Central ocular Surface Temperature (first 1 s post blink) in normal patients: 35.11 °C. Mean central cornea temperature after 5 s post blink in normal patients: 34.95 °C. Upper eyelid temperature in patients with meibomian gland dysfunction: 34.97 °C central, 35.40 °C nasal, 34.86 °C temporal. Upper eyelid temperature in patients with aqueous-deficient dry eye: 35.00 °C central, 35.44 °C nasal, 34.92 °C temporal. Ocular surface temperature in patients with meibomian gland dysfunction: 34.56 °C central, 35.12 °C nasal, 34.71 °C temporal. Ocular surface temperature in patients with aqueous-deficient dry eye: 34.56 °C central, 34.89 °C nasal, 34.71 °C temporal. Periorbital temperature in patients with meibomian gland: 35.63 °C superonasal, 35.07 °C inferonasal, 34.28 °C temporal. Periorbital temperature in patients with aqueous-deficient dry eye: 35.73 °C superonasal, 35.35 °C nasal, 34.58 °C temporal.
Purslow C, Wolffsohn JS [18]	NR	NR	Corneal temperature: 35.9 °C ± 0.7 °C. Ocular surface temperature: 32.9 °C–36 °C.
Klamann MKJ, Maier AKB, Gonnermann J, Klein JP, Pleyer U [30]	24 °C ± 1.50 °C	43.5% ± 3.0%	Ocular surface temperature in women: 33.86 °C ± 0.67. Ocular surface temperature in men: 34.15 °C ± 0.66.
Kohlmann H, Storch H, Lommatzsch PK [31]	18–20 °C	NR	Shows an increase of 0.9 °C in patients with infiltrative orbitopathy and immunosuppression compared to healthy individuals.
Morgan PB, Soh MP, Efron N [23]	Target environment temperature 20 °C.	NR	A decrease of less than 0.010 °C per year of age.
Kessel L, Johnson L, Arvidsson H, Larsen M [24]	Temperature was measured in three different rooms: typical room temperature (22–28 °C), a sauna (43 °C), and a cooling room (2 °C).	NR	Shows that corneal temperature stabilizes between 36.5 °C and 37 °C despite the increase in the body temperature.
Matteoli S, Favuzza E, Mazzantini L, Aragona P, Cappelli S, Corvi A, Mencucci R [44]	22.1 °C ± 0.5°C	43% ± 4%	Ocular surface temperature in patients with meibomian gland dysfunction: 34.74 °C central, 35.69 °C nasal, 34.83 °C temporal. Ocular surface temperature in patients with aqueous-deficient dry eye: 34.57 °C central, 35.46 °C nasal, 34.71 °C temporal.
Kamao T, Yamaguchi M, Kawasaki S, Mizoue S, Shiraishi A, Ohashi Y [45]	26.5 °C ± 1.5 °C.	42.5% ± 2.5%	Ocular surface temperature in patients with aqueous-deficient dry eye: 34.45 °C ± 0.86 central 34.96 °C ± 0.73 nasal conjunctiva 34.75 °C ± 0.82 temporal conjunctiva.
García-Porta N, Gantes-Nuñez FJ, Tabernero J, Pardhan S [46]	23 °C	45%	Shows that eyes with glaucoma cooled significantly faster (average decrease of 0.49 °C during the first second versus 0.24 °C in the control group).

**Table 2:** Thermal evaluation in patients with dry eye.

Region of Interest (Study)	Sample Size	Equipment	Meibomian Gland Dysfunction—Mixed Dry Eye (Evaporative and Aqueous-Deficient Dry Eye)	Aqueous-Deficient Dry Eye (ADDE)
Upper eyelid temperature (Abreau et al., 2016) [26]	10 in each group	Thermovision A40, FLIR System Inc. (Wilsonville, OR, USA)	34.97 central 35.40 nasal 34.86 temporal	35.00 central 35.44 nasal 34.92 temporal
Ocular surface temperature (Abreau et al., 2016) [1]	10 in each group	Thermovision A40, FLIR System Inc. (Wilsonville, OR, USA)	34.56 central 35.12 nasal 34.71 temporal	34.56 central 34.89 nasal 34.71 temporal
Ocular surface temperature (Matteoli et al., 2017) [44]	38	FLIR 320A (FLIR Systems, (Wilsonville, OR, USA)	34.74 central 35.69 nasal 34.83 temporal	34.57 central 35.46 nasal 34.71 temporal
Ocular surface temperature (Kamao et al., 2011) [42]	30	Ocular Surface Thermographer (TOMEY Corporation, Nagoya, Aichi, Japan)		34.45 ± 0.86 central 34.96 ± 0.73 nasal conjunctiva 34.75 ± 0.82 temporal conjunctiva
Periorbital temperature (Abreau et al., 2016) [26]	10 in each group	Thermovision A40, FLIR System Inc. (Wilsonville, OR, USA)	35.63 superonasal 35.07 inferonasal 34.28 temporal	35.73 superonasal 35.35 nasal 34.58 temporal
